# Relationship between the degree and direction of nasal septum deviation and nasal bone morphology

**DOI:** 10.1186/s13005-017-0136-2

**Published:** 2017-02-28

**Authors:** Ismail Serifoglu, İbrahim İlker OZ, Murat Damar, Mustafa Cagtay Buyukuysal, Alptekin Tosun, Özlem Tokgöz

**Affiliations:** 1 Department of Radiology, Bagcilar Training and Research Hospital, Istanbul, Turkey; 2 Department of Radiology, Bulent Ecevit University Faculty of Medicine, Zonguldak, Turkey; 3 Department of Head and Neck Surgery, Bulent Ecevit University Faculty of Medicine, Zonguldak, Turkey; 4 Department of Biostatistics, Bulent Ecevit University Faculty of Medicine, Zonguldak, Turkey; 5 Department of Radiology, Giresun University Faculty of Medicine, Giresun, Turkey; 6 Department of Radiology, Antalya Eğitim ve Araştırma Hastanesi, Antalya, Turkey

**Keywords:** Nasal bone, Nasal septum, Rhinoplasty, Septoplasty

## Abstract

**Background:**

Nasal septal deviation may affect nasal bone growth and facial morphology. Knowledge of nasal morphologic parameters may plays an important role in planning successful rhinoplasty and septoplasty operation. The aim of our study was to evaluate the relationship between the direction and degree of nasal septal deviation with nasal bone morphology, along with factors such as age and gender.

**Methods:**

Maxillofacial computed tomography (CT) of 250 patients with nasal septal deviation was analyzed retrospectively in this study. We excluded patients with factors that could affect their nasal bone morphology, and a total of 203 patients (111 males, 92 females; mean age, 36.23 years; age range, 18–79 years) were evaluated. The nasal deviation angle was measured on coronal CT images as the angle between the most deviated point of the septum, and the midline nasal morphology was determined by measuring nasal length, internasal angle and lateral and intermediate nasal thickness on both sides.

**Results:**

The deviation of nasal septum has been detected as to the right in 107 patients (52.7%) and to the left in 96 patients (47.3%). Lateral and intermediate nasal bone thickness and nasal bone length were significantly greater on the ipsilateral deviation side (Table 3). No significant correlation was found between the variation of the nasal deviation angle and nasal bone morphology (Table 4). There were significant differences between the sexes for all investigated parameters except for the nasal deviation angle (*p* = 0.660). We found that the only internasal angle increases with aging (*p* = 0.002).

**Conclusion:**

The study shows that the direction of nasal septal deviation may be a factor that affects nasal bone morphology.

## Background

The nasal septum is located in the medial portion of the nasal cavity and it is on the major part of the nose structure. It is divided into a posterior part by the vomer and perpendicular plate of the ethmoid bone and an anterior part by the quadrangular cartilage [1, 2].

Nasal septum deviation is the most common anatomic variation in up to 80% of healthy adults [3, 4]. When we consider healthy nasal respiration the anatomical and morphological characteristics of the bony and cartilaginous parts of the nasal septum play an important role. Nasal septal deviation is also linked to sleep apnea, repetitive sneezing, nosebleeds, sinusitis and difficulty breathing [1]. Some investigators linked the sense of smell sense with septal deviation.

The nasal septum may affect nasal bone growth and facial morphology. From the initial growth stages, the maxillary bone and nasal structure have significant anatomic connections because of their close embryologic development [5]. In the growth period, the nasal septum acts as a growth plate that affects surrounding bones and facial skeletal tissues [6]. Thus, nasal septum deviation affects facial morphologic parameters such as interalveolar distance and maxillary rotation distance, causing compensatory changes in the lateral nasal wall and septal deviation, which are associated with nasal floor and palatal region asymmetries [1, 6, 7]. The severity of septal deviation also affects the ipsilateral lateral lamina of the cribriform plate width and ipsilateral middle turbinate length [8].

Some morphological characteristics of the nose such as bone length and thickness may present different forms according to factors correlated with age, gender, climate and race [9–11]. Morphologic features of the nasal bone play an important role in planning successful septoplasty and rhinoplasty surgeries. The chose between open rhinoplasty approach and endonasal approaches are commonly based on morphologic parameters. The information about each parameter during rhinoplasty, allows minimizing postoperative complication such as distortion, tissue edema and hemorrhage [12]. The effect of nasal septum deviation on nasal morphologic parameters such as thickness and length was not investigated. The aim of our study was to evaluate the relationship between the direction and degree of nasal septum deviation with nasal bone morphology, along with factors such as age and gender.

## Methods

### Study population

Two hundred fifty patients with a nasal septum deviation who underwent maxillofacial CT between February 2013 and March 2015 were included retrospectively and randomly from archives of Bülent Ecevit University, Faculty of Medicine. Only adults over 18 years of age were included in the study. Patients’ medical records were investigated, and patients with a history of rhinoplasty, cranial and facial trauma or bone deformity (e.g. S-shaped septum deviation), and patients with a mass in the nasal cavity were excluded from the study. Two hundred three patients (111 male, 92 female; mean age, 36.23 years; age range, 18–79 years) were included. Our study protocol was conformed according to the 2013 Declaration of Helsinki and this study was approved by ethics committee of Bulent Ecevit University Medical School with approval number 2015-46-09/06.

**Fig. 1:**
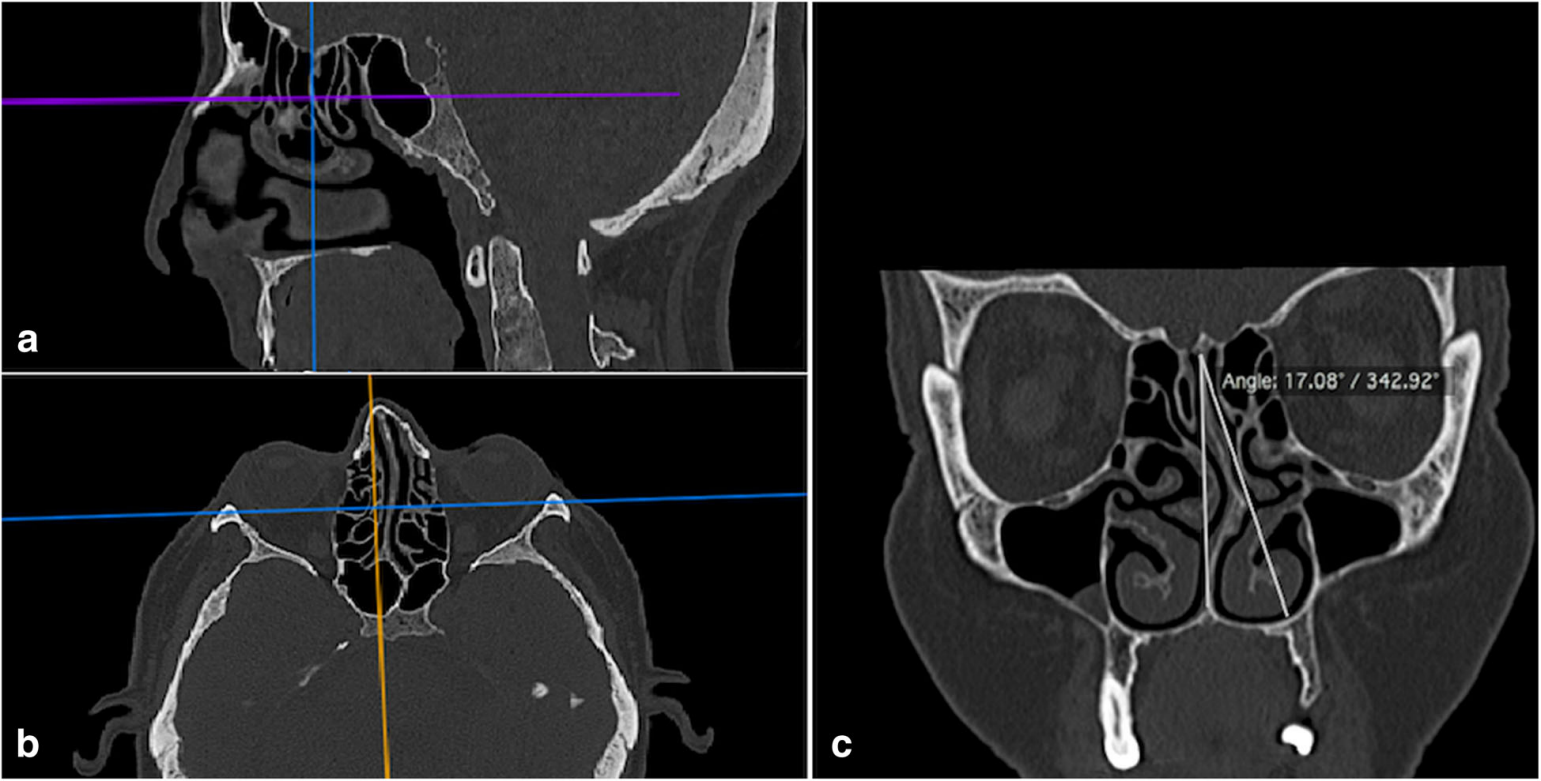
The reference lines on axial and sagittal MPR images was used for correction of measurement plane (**a**, **b**), the nasal deviation angle was measured on coronal MPR images as the angle between the most deviated point of the septum and the midline (**c**)

CT examinations were performed using an Activion 16 CT Scanner (Toshiba Medical Systems, 2008 Japan). The CT parameters were 120 kVp, 100–150 mA, 0.5 mm contiguous axial slice thickness, 512 × 512 matrix size, and field of view (FOV) of 240. The images were obtained in a supine position without rotation, flexion or extension. To solve asymmetry problem arising from patients malposition, the reference lines was used for correction of measurement plane in all patients (Figs. 1a, b and 4a, b). Multiplanar reconstructed (MPR) coronal and sagittal images were generated on a personal computer using OsiriX software (http://www.osirix-viewer.com). All measurements were performed by two radiologists with 9 and 7 years of experience in maxillofacial radiology in same software.

### Data collection

The nasal deviation direction was described by the convexity of the septal curvature. The nasal deviation angle was measured on coronal CT images as the angle between the most deviated point of the septum and the midline (Fig. 1). The line from the crista galli to the palatum was defined as midline [13]. Patients were divided into three groups according to the deviation angle: mild (<9°), moderate (9–15°), and severe (≥15°) [14, 15].

Nasal bone morphology was assessed by measuring the lateral and intermediate nasal bone thickness, nasal bone length and internasal angle.

**Fig. 2:**
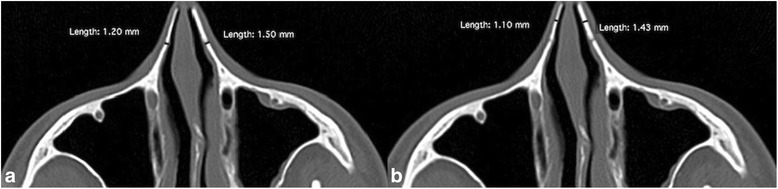
Nasal bone thickness measurement. **a** Lateral nasal bone thickness. **b** Intermediate nasal bone thickness measurement

The nasal bone thickness was measured in axial images at the site of the lateral and intermediate osteotomy lines. The lateral osteotomy lines run along nasomaxillary suture and intermediate (or midline) osteotomy lines runs along the internasal sututre. The lateral nasal bone thickness was measured at the nasomaxillary suture. Intermediate nasal bone thickness was measured at the midpoint between the nasomaxillary suture and the rhinion (Fig. 2) [9].

**Fig. 3:**
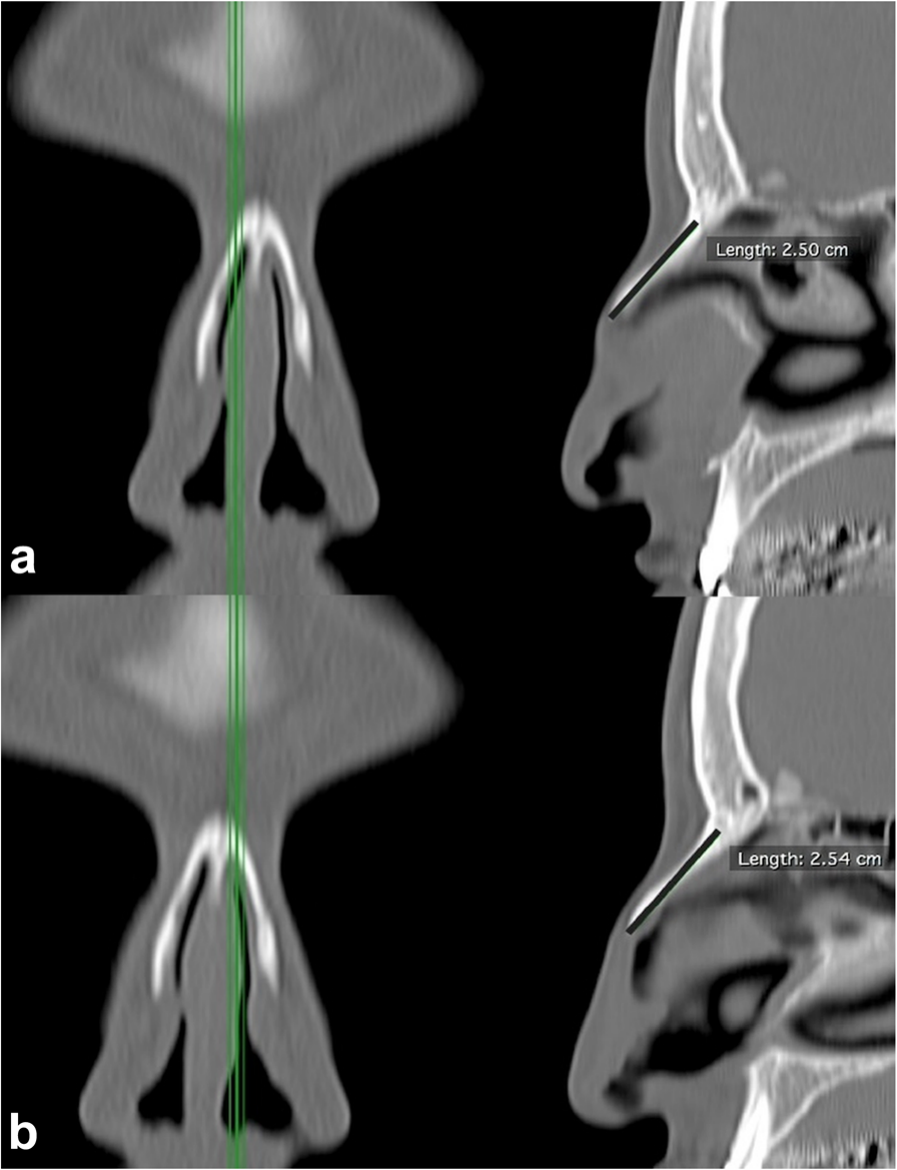
Measurement of the nasal bone length at the right (**a**) and the left side (**b**)

**Fig. 4:**
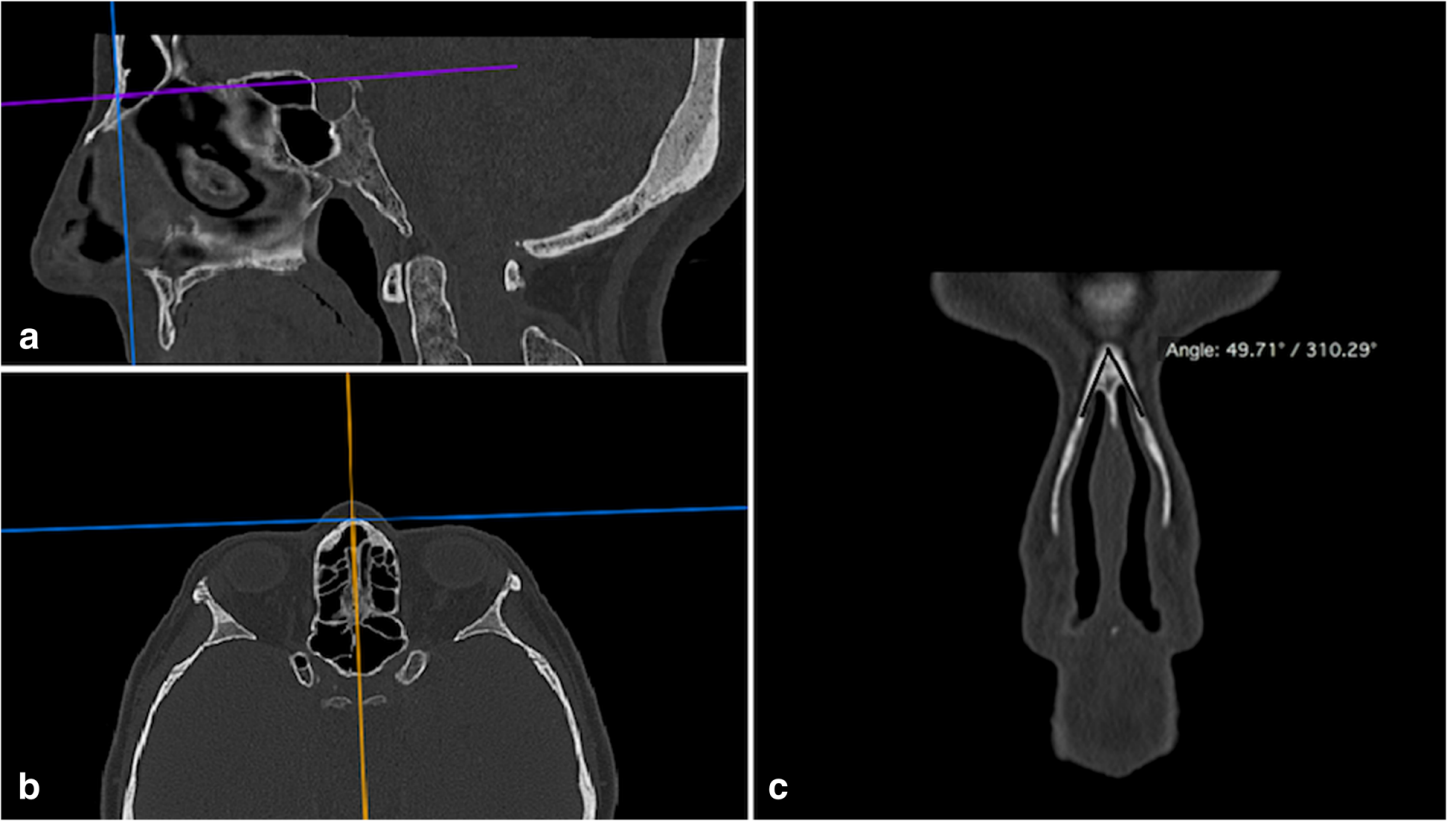
The reference lines on axial and sagittal MPR images was used for correction of measurement plane (**a**, **b**), the internasal angle was measured on coronal images after the x and y planes were brought to the nasion point (**c**)

The nasal bone length was measured from frontonasal suture to the endpoint of the nasal bone on the sagittal plane (Fig. 3) [16]. The internasal angle was measured on coronal MPR images at the site of the nasion point (Fig. 4) [10]. All parameters were measured bilaterally except internasal angle.

### Statistical analysis

Statistical analysis was performed using SPSS software for Windows (version 19.0, SPSS Inc., Chicago, IL, USA). Categorical variables are given as frequencies and percentages, and continuous variables are given as the mean, standard deviation, median, minimum and maximum values. The Shapiro Wilk test was used as a test of normality. The independent sample *t*-test and analysis of variance (ANOVA) were used for two- and three parametric group comparisons, and the Mann-Whitney U and Kruskal Wallis tests were used for two- and three non-parametric group comparisons. The Student’s *t*-test was used in intergroup comparison of parameters with normal distribution. For all statistical comparisons, a p value below 0.05 was considered statistically significant.

## Results

There were 107 patients in our study (52.7%) who had nasal septum deviation to the right, and 96 (47.3%) who had deviation to the left. No significant difference was found between deviation direction groups with regard to age, gender or deviation angle (*p* = 0.391, 0.325, 0.407 respectively; Table 1).

**Table 1:** Relationships between nasal deviation side and age, sex and nasal deviation angle

	Right* (*n* = 107)	Left* (*n* = 96)	*p* ^#^
Age	32 (18–79)	34.5 (18–79)	0.391
Nasal deviation angle	13.1 (5.1–29.2)	14.05 (4.9–34.1)	0.407
Sex			0.325

*Values are presented as the median (min-max)

^#^ *P* values were calculated using the Mann-Whitney *U* test

There were statistically significant differences between the sexes for all investigated parameters except for the nasal deviation angle (*p* = 0.660) (Table 2).

**Table 2:** Relationships between sex, nasal morphology and nasal deviation angles

	Male* (*n* = 111)	Female* (*n* = 92)	*p* ^#^
Nasal deviation angles (degree)	13.88 (4.9–31.2)	14.18 (5.1–34.1)	0.660
Internasal angles (degree)	53.08 (40.2–75.3)	50.25 (33.1–65.5)	0.020
Right LT (mm)	1.9 (1.3–2.93)	1.8 (1.15–2.66)	0.018
Left LT (mm)	1.9 (1.32–2.89)	1.8 (1.23–2.58)	0.012
Right IT (mm)	1.6 (1.06–2.67)	1.49 (1.08–1.93)	0.002
Left IT (mm)	1.61 (1.11–2.53)	1.47 (1.05–2.13)	<0.001
Right NBL (mm)	22.9 (14.29–32.62)	21.05 (12.58–29.33)	<0.001
Left NBL (mm)	22.62 (15.15–33.5)	20.74 (12.97–28.83)	<0.001

*LT* lateral nasal bone thickness, *IT* intermediate nasal bone thickness, *NBL* nasal bone length

*Values are presented as the median (min-max)

^#^ *P* values were calculated using the Mann-Whitney *U* test

Nasal bone deviation angles ranged between 4.9° and 34.1°. Mean deviation angles were 13.6 ± 5.29° for right deviation and 14.44 ± 6.08° for left deviation. The deviation angle indicated that 43 patients had mild deviation (Group 1), 77 patients had moderate deviation (Group 2) and 83 patients had severe nasal septal deviation (Group 3).

The median (min-max) value of the nasal deviation angle, lateral and intermediate bone thicknesses and length of nasal bone on the ipsilateral and contralateral are presented in Table 3. There were statistically significant differences between the ipsilateral and contralateral nasal septal deviation in all morphologic parameters except for the internasal angle (*p* = 0.283).

**Table 3:** Relationships between nasal morphology and nasal deviation side

	Ipsilateral* (*n* = 107)	Contraletral* (*n* = 96)	*p* ^#^
Internasal angle (degree)	52.7 (33.1–75.3)	51.4 (38.7–72.8)	0.283
Right LT (mm)	1.93 (1.34–2.93)	1.76 (1.24–2.65)	0.002
Left LT (mm)	1.96 (1.43–2.79)	1.79 (1.23–2.89)	<0.001
Right IT (mm)	1.62 (1.11–2.68)	1.51 (1.06–2.20)	0.002
Left IT (mm)	1.65 (1.18–2.25)	1.48 (1.05–2.53)	<0.001
Right NBL (mm)	22.6 (14.85–32.62)	21.41 (12.58–31.07)	0.027
Left NBL (mm)	22.53 (13.29–33.5)	21.28 (12.97–29.33)	0.025

*LT* lateral nasal bone thickness, *IT* intermediate nasal bone thickness, *NBL* nasal bone length

*Values are given as the median (min-max)

^#^ *P* values were calculated using the Mann–Whitney *U* test

There were no significant differences in the internasal angle, nasal bone length and lateral and intermediate bone thicknesses between nasal septal deviation angle groups (Table 4).

**Table 4:** Relationships between nasal morphology and nasal deviation angle groups

	Group I* (0°–9°)	Group II* (9°–15°)	Group III* (≥15°)	*p* ^#^
Internasal angle (degree)	52.8 (33.1–75.3)	51.1 (40.2–69.7)	52.4 (38.4–69)	0.662
Right LT (mm)	1.82 (1.43–2.93)	1.8 (1.24–2.59)	1.9 (1.25–2.86)	0.265
Left LT (mm)	1.84 (1.32–2.89)	1.86 (1.23–2.54)	1.89 (1.27–2.71)	0.650
Right IT (mm)	1.51 (1.07–2.68)	1.55 (1.06–2.32)	1.57 (1.06–2.31)	0.442
Left IT (mm)	1.5 (1.14–2.53)	1.61 (1.15–2.42)	1.57 (1.05–2.21)	0.151
Right NBL (mm)	22.09 (13.95–32.62)	22.35 (15.8–31.1)	21.87 (12.5–30.34)	0.821
Left NBL (mm)	21.62 (12.97–29.25)	22.42 (13.7–33.5)	24.54 (13.3–30.7)	0.519

*LT* lateral nasal bone thickness, *IT* intermediate nasal bone thickness, *NBL* nasal bone length

*Values are given as the median (min-max)

^#^ *P* values were calculated using the Mann-Whitney *U* test

When we investigated the relationships between age and bone morphology, only internasal angle was increased with aging (*p* = 0.002).

## Discussion

Nasal septum deviation can disturb nasal physiology, and it can be combined with conchal hypertrophy or other anatomical variations. Nasal septum deviation can narrow the middle meatus by pushing the concha laterally. Besides nasal obstruction, nasal septum deviation exerts pressure on neighboring structures. This, in turn, disturbs drainage pathways, affects mucosal ciliary function through contact, and leads to obstruction and secondary nasal infection in all sinuses by disturbing normal mucus drainage. These mucosal abnormalities were most frequently noted in the maxillary sinus region [14]. The anatomical integrity and functional capability of the septum may allow the operation of two sides of the nose, where each of them have separate vascular support and innervation. The operation of two separate airways, rather than a single unified airway, provides some advantages for conditioning the air and respiratory defense.

Our study shows that in patients with nasal septum deviation, the ipsilateral nasal bone length and bone thicknesses were greater than that on the contralateral side. It is thought that nasal septum deviation affects nasal bone development because this close relationship originates from embryologic stages of development [5]. Studies have suggested that there is a significant facial growth delay on the concave side of the deviated nose [17]. Our data support this finding that nasal bone thickness was smaller on the contralateral side of a deviation. Significant relationships could not be obtained between the size of the septal area and the degree of septal deviation [18], on the other hand in previous research the association between nasal bone and septal deviation has not been studied. In this study, the nasal length and nasal bone thickness were affected by the side of the septal deviation, but this was not related to the degree of septal deviation.

Currently, nasal osteotomies are performed with mechanical force and thus, they lead to large amounts of trauma to the nasal mucosa, which may contribute to extended post-operative ecchymosis. Fragmented fracture of the nasal bones may also lead to a negative cosmetic outcome [19, 20]. We believe that complications can be reduced by choosing the thinnest side of the nasal bone during an osteotomy, based on nasal septal deviation. Also identification of, and intervention in, the long side of the nasal bone according to the nasal septum deviation may lead to better and proper healing of nasal asymmetry.

Some studies focused on the effect of the degree of nasal septum deviation on the surrounding structures. Kapusuz Gencer et al. reported that severe nasal septum deviation influences maxillary sinus volume [14]. Poorey et al. demonstrated that there was no significant relationship between nasal septum deviation angle and sinusitis in mild to severe degree septum deviation [7]. Nomura et al. did not find a significant correlation between nasal bone overlap and degree of nasal septum deviation [18]. Similarly, in our study, there was no significant difference in nasal bone morphology parameters between a mild and severe degree of septal deviation.

The nasal bone length and bone thicknesses measured in our study were significantly greater on either side in male patients. Our results support the differences in nasal bone morphology between the sexes, as reported in previous studies. In a study by Yüzbaşıoğlu et al. using three-dimensional reconstructed images to assess the morphology of the nasal bone and the piriform aperture, nasal bone length on both sides and on the center line was significantly greater in males than in female patients [10]. In a study by Karadağ et al. on the Anatolian population, no significant difference was found between the sexes in nasal bone thickness, although nasal bone length was found to be greater in males [16]. In a study of nasal bone morphology in Koreans, Hwang et al. reported a statistically significant difference between the sexes in terms of nasal bone height [11].

Aging causes bone resorption especially in the middle third of the face. This is manifested by enlargement of the superomedial and inferolateral orbital aperture width [12]. Our results are consistent with this data, which indicates enlargement of the internasal angle with aging. This is linked with bone resorption and also with weakened, and loss of, nasal bone support [21]. Our findings emphasize that the bony elements of the midface change to a great extend with aging and that the bony aging process is primarily that of contraction and deterioration and not of expansion. This outcome may help surgeon to understand changes in facial skeleton with aging, and in facial rejuvenation surgery soft tissue augmentation may be a better approach compared with bony augmentation, because the platform underneath the bony implant may deteriorate over time [21].

## Conclusion

Our study shows that nasal septum deviation may be a factor affecting nasal bone morphology. We evaluated the deviated side, and found that nasal bone length and thickness on the deviated side were significantly greater than on the contralateral side. However, when an evaluation was made according to septum deviation angles, there was no morphological difference between the groups. Our study also shows that sex influences the nasal morphologic parameters, but aging was limited effect on the morphologic data.
